# The effect of positive autobiographical memory retrieval on decision-making under risk: A computational model-based analysis

**DOI:** 10.3389/fpsyt.2022.930466

**Published:** 2022-09-06

**Authors:** Natsumi Shimizu, Yasuhiro Mochizuki, Chong Chen, Kosuke Hagiwara, Karin Matsumoto, Yusuke Oda, Masako Hirotsu, Emi Okabe, Toshio Matsubara, Shin Nakagawa

**Affiliations:** ^1^Division of Neuropsychiatry, Department of Neuroscience, Yamaguchi University Graduate School of Medicine, Ube, Japan; ^2^Center for Data Science, Waseda University, Tokyo, Japan

**Keywords:** positive autobiographical memory, decision-making, risk preference, probability weighting, computational neuroscience

## Abstract

Psychiatric disorders such as depressive and anxiety disorders are associated with altered decision-making under risk. Recent advances in neuroeconomics and computational psychiatry have further discomposed risk-based decision-making into distinct cognitive computational constructs and showed that there may be disorder-specific alterations in these constructs. As a result, it has been suggested these cognitive computational constructs may serve as useful behavioral biomarkers for these disorders. However, to date, little is known about what psychological or behavioral interventions can help to reverse and manage the altered cognitive computational constructs underlying risk-based decision-making. In the present study, we set out to investigate whether recalling positive autobiographical memories may affect risk-based decision-making in healthy volunteers using a description-based task. Specifically, based on theories of behavioral economics, we dissected risk preference into two cognitive computational constructs, utility sensitivity and probability weighting. We found that compared to recalling neutral memories, retrieving positive autobiographical memories increased utility sensitivity (Cohen's *d* = 0.447), indicating reduced risk aversion. Meanwhile, we also tested the influence of memory retrieval on probability weighting, the effect, however, was unreliable and requires further in-depth investigation. Of clinical relevance, the change in risk aversion after recalling positive memories was in the opposite direction compared to those reported in psychiatric disorders. These results argue for the potential therapeutic effect of positive autobiographical memory retrieval for the amendment of altered risk-based decision-making in psychiatric disorders.

## Introduction

Psychiatric disorders such as depressive and anxiety disorders are associated with deficits in cognition and decision-making (1–4). For instance, both depressive and anxiety disorders have been characterized by excessive risk aversion or avoidant behaviors (5, 6). In behavioral economics, risk is defined as the variability of outcome and people's attitude toward risk is called risk preference. Preferring outcomes with high certainty (e.g., $50 guaranteed over 50% chance of getting $100) is said to be risk-averse, while the opposite is called risk-seeking and having no preference is known as risk-neutral. Among these three preferences, risk-neutrality is considered rational and characterizing an economic man. Importantly, excessive risk aversion has been considered a key contributor to the maintenance and recurrence of depressive and anxiety disorders (3, 7, 8).

To provide a mechanistic account of decision-making, recent advances in neuroeconomics and computational psychiatry have further discomposed risk-based decision-making into multiple, distinct cognitive computational constructs (9, 10). For instance, two commonly studied constructs of risk preference are utility sensitivity and probability weighting. In standard expectation-based theories in economics, risk preference is captured by a utility or utility sensitivity function (e.g., a power function). Here, a linear utility function indicates risk-neutrality, a concave utility function indicates risk aversion, and a convex function indicates risk-seeking. Most individuals are considered to have a concave utility function (i.e., being risk-averse) because of the law of diminishing marginal utility (11). Nevertheless, people tend to have different risk preferences at small vs. large probabilities. To account for this phenomenon, Prospect theory (12, 13) introduced probability weighting function. Thus, most individuals tend to overweight small probabilities (i.e., risk seeking) and underweight large probabilities (i.e., risk aversion), as indicated by an inverse-S-shaped, non-linear probability weighting function.

Using this computational model-based framework, it has been reported that generalized anxiety disorder is associated with a more concave utility function [indicating risk aversion, (14)]. In contrast, obsessive-compulsive and hoarding disorders (15) and depression (16) are associated with an altered probability weighting function, in which patients with obsessive-compulsive and hoarding disorders and individuals with more depressive symptoms tend to underweight small probabilities and overweight large probabilities compared to healthy subjects. These disorder-specific changes argue for the usefulness of the cognitive computational constructs underlying risk-based decision-making as behavioral biomarkers of these disorders.

Despite these fruitful progresses made by recent research, little is known about what psychological, behavioral, or dietary interventions can help to reverse and manage the altered cognitive computational constructs underlying risk-based decision-making in psychiatric patients. In the present study, therefore, to provide insights into the development of effective interventions that may help to treat decision-making impairments in clinical patients, we conducted a randomized controlled crossover experiment with healthy volunteers to investigate if recalling positive autobiographical memories may affect risk-based decision-making and its cognitive computational constructs. Here, to evaluate risk-based decision-making, we used a description-based task in which subjects were given explicit information on reward magnitude and probability. The reason we focused on positive autobiographical memory retrieval was that it activates the midbrain dopaminergic reward system, including the striatum and the medial prefrontal cortex (17–19). The latter has been suggested to be the neural substrate of risk preference and probability weighting (20–22).

## Materials and methods

### Participants

The study was approved by the Institutional Review Board of Yamaguchi University Hospital and preregistered on the University hospital Medical Information Network Clinical Trial Registry (UMIN-CTR, register ID: UMIN000044704). Thirty-four healthy subjects were recruited *via* posters placed on campus and through word-of-mouth. This sample size was similar to previous studies (18) and considered appropriate according to a priori power analysis (to detect a moderate effect size of *d* = 0.50 using a within-subject design, with a power of 0.8, alpha = 0.05, two-sided, 34 subjects were required). One subject dropped out because of being sick on the scheduled experimental day, leaving thirty-three subjects for the final analysis (18 males, 15 females, age: 21.18 ± 0.98 years, all were undergraduate students).

The study was carried out according to the Declaration of Helsinki. All subjects provided written informed consent. To remove the influence of age, we limited subjects to those in their twenties. The exclusion criteria were reporting any current memory or mental disorders (or currently seeking medical examinations due to suspicion of these disorders), being unable to retrieve 20 or more items of positive and neutral memories, respectively, in the autobiographical memory recall test (see below), and being judged to be unsuitable as a subject due to other issues. No subject was excluded because of meeting any of these exclusion criteria.

### Procedure and design

**Figure 1:**
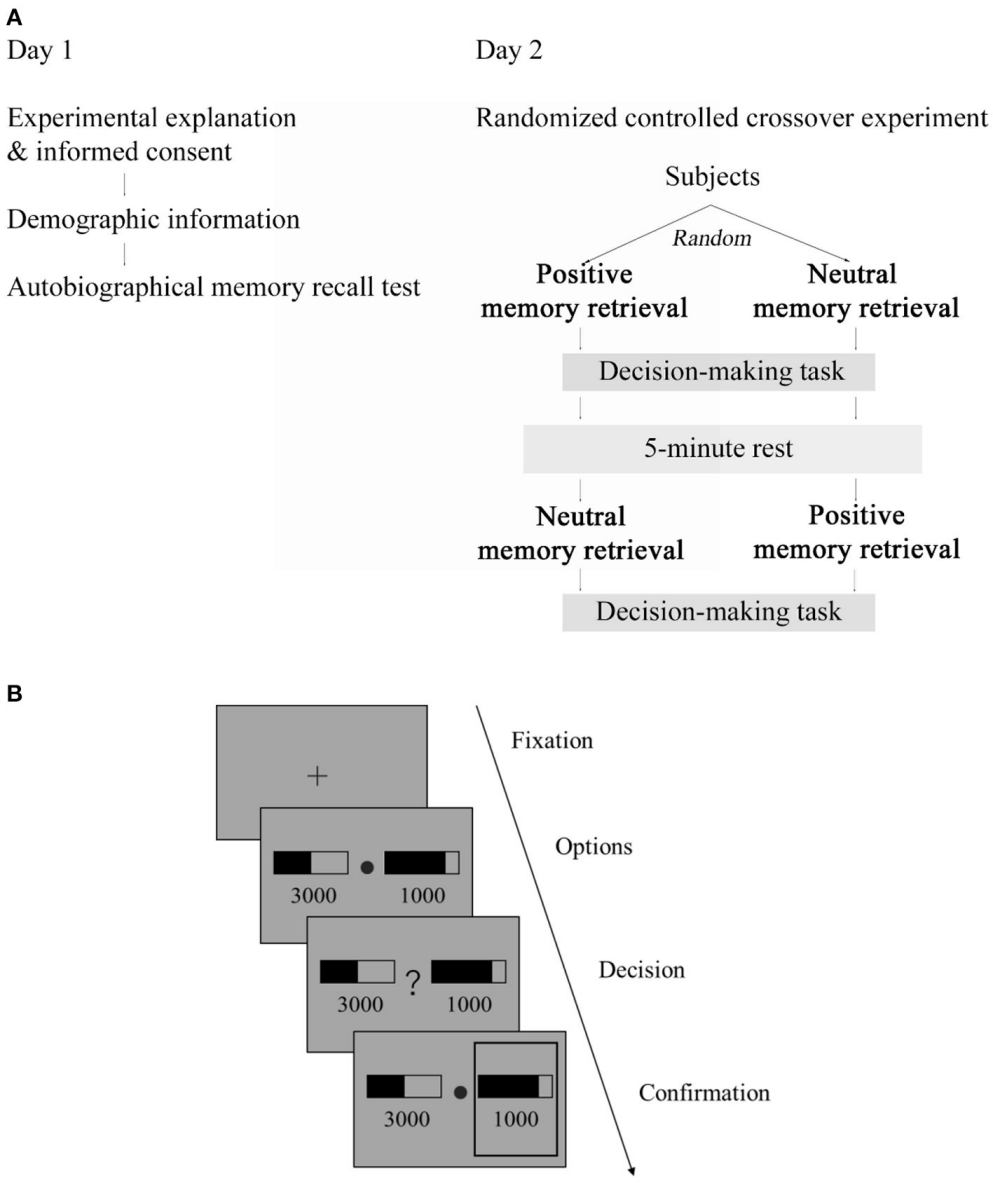
Schematic illustration of the procedure and decision-making task. **(A)** Procedure, the study was conducted on two separate days: on day 1, subjects performed an autobiographical memory recall test; on day 2, they were assigned to receive memory retrieval intervention in a randmozed controlled crossover experiment. **(B)** Illustration of the decision-making task. After a fixation phase, two gambling options, each consisting of a reward magnitude (in JPY) and the probability of receiving that magnitude of reward (indicated by a black bar), were shown. A question mark then occurred in the center and subjects were asked to choose one option that maximized their reward. The chosen option was highlighted by a gray frame.

The study was conducted on two separate days (Figure 1A). On day 1, subjects provided written informed consent after receiving a detailed description of the study and filled out demographic information. They were explained that the objective of the study was to investigate the effect of positive autobiographical memory retrieval on decision-making. They then conducted an autobiographical memory recall test, in which, given 87 common life event cues (e.g., getting an acceptance letter), subjects selected memories in which they had been personally involved. The 87 common life event cues were created based on previous studies (18, 23) and our pilot testing. For each selected memory, subjects were asked to recall only positive or neutral events and then give a brief description and indicate the location and date of the event. For the brief description, they were asked to be specific so that they could easily recall the event later upon reading the description. Furthermore, they also rated the valence (neutral or positive) and emotional intensity (1–4: 1 = not intense, 4 = very intense) of the memory as well as how they felt when recalling the memory (1–4: 1 = neutral, 4 = very good). In preparation for the day 2 intervention session, twenty of each subject's memories with a positive valence and the highest combined emotional intensity and feeling ratings (the sum of the two) were selected as positive memories. Similarly, twenty of each subject's memories with a neutral valence and the lowest combined emotional intensity and feeling ratings were selected as neutral memories.

Subjects returned for the main experimental session on another day (i.e., day 2) within a week. Before the day 2 laboratory visit, subjects were instructed to get enough sleep on the previous night and refrain from engaging in intensive physical activities, smoking, and drinking coffee and energy drinks for at least 2 h before coming to the laboratory visit. They were also asked to reschedule the experiment if they were sick or did not feel well on the experimental day. Upon arriving, subjects first answered questions to confirm whether they adhered to the above instructions and then filled out the Positive and Negative Affect Schedule [PANAS, (24)] indicating their baseline mood. PANAS measures mood at the moment in terms of positive affect and negative affect using 10 words that describe feelings. Each word was rated on a scale of 1 (“not at all”) to 6 (“extremely”). Based on PANAS, no subject at the time had extremely high positive or negative mood (positive affect: range 11–41; negative affect: range 10–32).

We used a within-subjects randomized controlled crossover design for the main study (Figure 1A). Subjects were randomly assigned to receive the experimental and control interventions in a counterbalanced order and immediately after each intervention, they conducted a decision-making task. The above 20 positive memories were used for the experimental intervention, and the 20 neutral memories was used for the control intervention. Given that the decision-making task (120 trials, see below) we employed here took about 15 min and typically required one short break amid, we split the intervention and decision-making task into two sessions. Thus, in session 1, subjects first recalled 10 memories, after which they conducted 60 trials of the decision-making task. After a short break of about 1 min, in session 2, subjects recalled the remaining 10 memories and then conducted the remaining 60 trials of the decision-making task. For data analysis, nevertheless, we combined the data of the two sessions.

For each memory, subjects were shown the initial cue together with their written responses in the day 1 autobiographical memory recall test (see Supplementary Figure S1 for an example) for 14 s. During this period, they were asked to recall and elaborate on the memory for 14 s silently. Following previous studies (17–19), for each memory, subjects indicated the valence and emotional intensity of the memory and reported how they felt when recalling the specific memory (4 s each).

During the memory retrieval (for both experimental and control interventions), subjects' heart rate (HR) and heart rate variability (HRV) were monitored using an Apple Watch Series 4 (Apple Inc.), the accuracy of which has been validated (25, 26). Immediately after memory retrieval in each section, subjects indicated their present mood in terms of pleasure, relaxation, and vigor using a visual analog scale (27). For data analysis of HR, HRV, and mood, the average of the two sessions was used. After the first phase of the intervention and decision-making task, subjects rested for 5 min as a washout period.

### Decision-making task

We adapted the decision-making task used by Hsu et al. (20). In this task (Figure 1B), given two gambling options each consisting of a reward magnitude (in Japanese yen) and reward probability (in percentage indicated by a black bar), subjects were asked to choose the one that maximized the reward they receive. We used the exact stimuli of reward magnitude and probability generated by 17 but multiplied the magnitude by 100 to reflect the exchange rate (from dollars to Japanese yen).

After an inter-trial interval or fixation phase of 1.5 s, the options were presented for 3 s. After a question mark occurred in the center, subjects had to indicate their choice by pressing one of two predefined keys within 3 s. The chosen option was then highlighted by a gray frame. Subjects were told that failing to respond within 3 s would be treated as no response and they could get no reward on that trial.

### Computational modeling of the decision-making behavioral data

As shown in Table 1, we fitted four models to simulate subjects' choice behaviors. One was based on the standard value function in which magnitude was multiplied by probability. The other three further introduced a non-linear utility function (i.e., a power function) and/or a non-linear probability weighting function [the one-parameter Prelec weighting function, (28)]. λ and γ are the parameters of utility sensitivity and probability weighting, respectively. Subjects were modeled to choose between two options according to their value difference based on the softmax rule whose stochasticity was controlled by an inverse temperature parameter β.

**Table 1:** Model specification and fitting results.

**Model No**.	**Model description**	**Equation**	**Free parameters**	**Bayesian hierarchical expectation-maximization**		**Maximum likelihood**	
				**iBIC Neutral**	**iBIC Positive**	**AIC Neutral**	**AIC Positive**
1	Linear utility and linear probability weighting	*V*(*X*) = *rp*	β	5,206.8	5,180.9	157.07	156.22
2	Non-linear utility and linear probability weighting	*V*(*X*) = *r*^λ^*p*	λ,β	5,025.5	5,038.5	108.85	120.06
3	Linear utility and non-linear probability weighting	*V*(*X*) = *re*^−(−log*p*)^^γ^	γ,β	3,672.9	4,086.6	151.43	150.32
4	Non-linear utility and non-linear probability weighting	*V*(*X*) = *r*^λ^*e*^−(-log*p*)^^γ^	λ,γ,β	3,527.8	3,907.8	102.63	112.30

Smaller iBICs and smaller AICs indicate better model fits. Neutral, neutral autobiographical memory retrieval. Positive, positive autobiographical memory retrieval. r indicates reward and p indicates probability.

To fit the models to subjects' choices, we used a Bayesian hierarchical expectation-maximization method (6, 29). In brief, given a current estimate of group-level prior distribution for each model parameter, we randomly sampled 100,000 sets of parameters and used the resulting likelihoods as importance weights to update the current prior distributions. This procedure was repeated iteratively until the estimate of model evidence stopped increasing. We then estimated the parameters for each subject as a weighted mean of the final 100,000 parametrizations. The prior distributions for β, λ, and γ were modeled as gamma distributions and were initialized to support wide ranges of possible values.

We compared different models using the integrated Bayesian Information Criterion (iBIC), which penalizes the sum of model evidence for each subject by the number of parameters and the number of choices made (30). Smaller iBIC values indicate more parsimonious model fits. As can be seen from Table 1, the fourth model incorporating both non-linear utility and non-linear probability weighting had the smallest iBIC and was the winning model. Parameters estimated from this model, therefore, were used for subsequent analysis.

In addition to the Bayesian hierarchical expectation-maximization method with population-level priors, we also tested a commonly used individual-level fitting method without population-level priors, the maximum likelihood method, which was executed with the Matlab command “fmincon”. For model selection, the Akaike Information Criterion (AIC) was employed. Smaller AICs indicate more parsimonious model fits. Similar to iBIC, the fourth model incorporating both non-linear utility and non-linear probability weighting was the winning model.

### Statistical analysis

MATLAB2018b and IBM SPSS Statistics 26.0 were used for statistical analysis. The normality of the data was checked using the Shapiro–Wilk test. Paired *t*-tests or Wilcoxon signed-rank tests were used to compare differences between interventions. G^*^Power Version 3.1.9.7 (31) was used to estimate effect sizes (Cohen's *d*). A significance level of *p* < 0.05 was used.

## Results

### Memory and feeling ratings, mood, HR, and HRV

**Figure 2:**
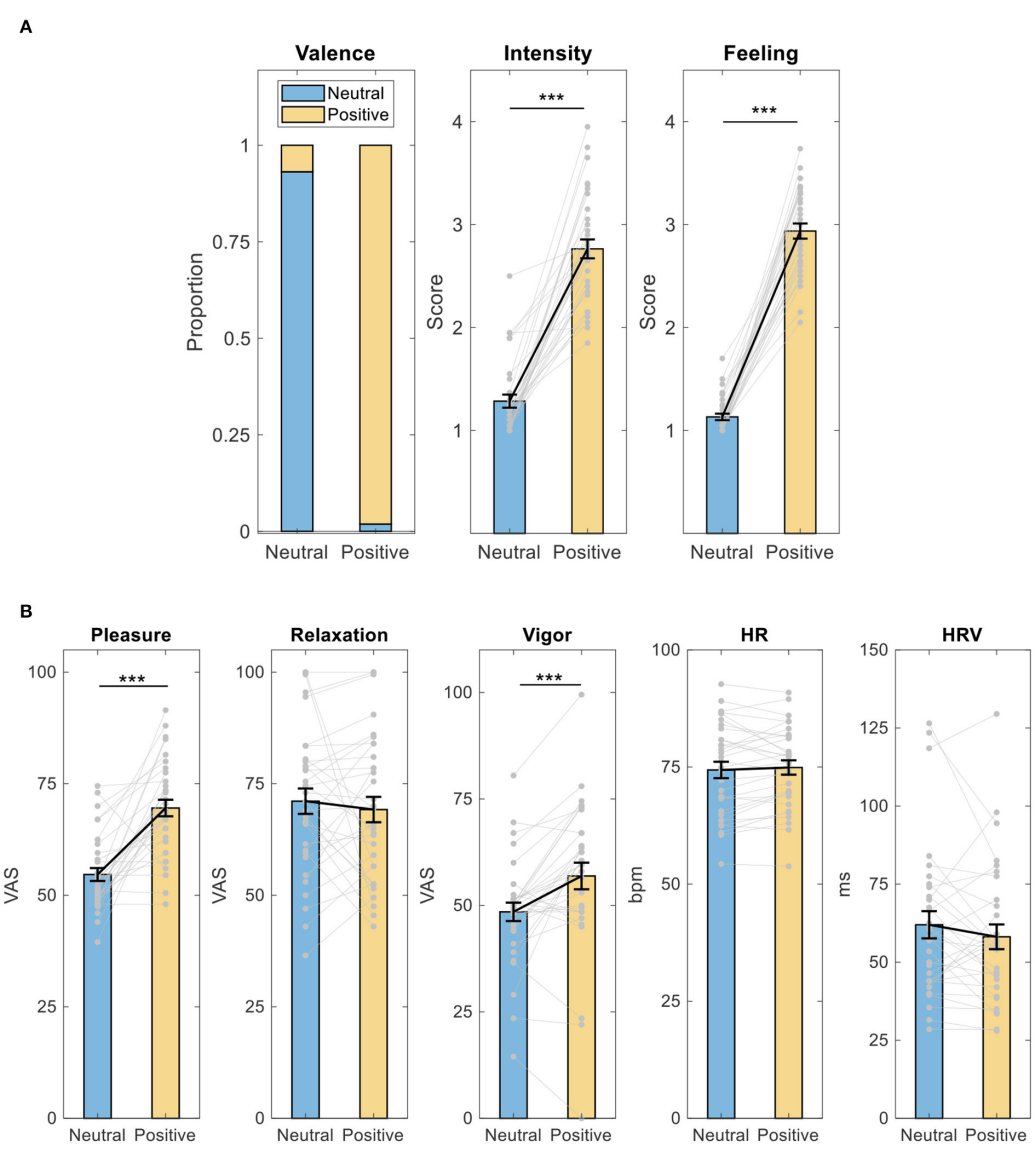
Intervention effect on memory and feeling ratings, mood, HR, and HRV. **(A)** Memory and feeling ratings; **(B)** mood, HR, and HRV. ***p < 0.001, paired *t*-test. HR, heart rate. HRV, heart rate variability; VAS, visual analog scale; bpm, beat per minutes; ms, milliseconds. Data shown as mean ± SE.

During neutral memory retrieval, subjects on average endorsed 93.14% of the memories to be neutral during neutral memory retrieval and endorsed 98.14% of the memories to be positive during positive memory retrieval (Figure 2A). Meanwhile, compared to neutral memory retrieval, subjects rated memories during positive memory retrieval as being more intense (paired *t*-test, *t* = −12.755, *p* = 4 × 10^−14^, *d* = 2.22) and feeling better (paired *t*-test, *t* = −21.301, *p* = 2 × 10^−20^, *d* = 3.71).

Furthermore, as shown in Figure 2B, compared to neutral memory retrieval, subjects reported feeling more pleasant (paired *t*-test, *t* = −6.696, *p* = 1 × 10^−7^, *d* = 1.17) and vigorous (paired *t*-test, *t* = −4.101, *p* = 3 × 10^−4^, *d* = 0.714) after positive memory retrieval. There was no difference in feelings of relaxation (Wilcoxon signed-rank test, *Z* = −0.009, *p* = 0.993, *d* = −0.115), HR (paired *t*-test, *t* = −0.795, *p* = 0.433, *d* = 0.141), or HRV (paired *t*-test, *t* =1.359, *p* = 0.184, *d* = −0.240).

Consistent with previous studies (17, 18), these results suggest that the protocol of memory retrieval is reliable.

### Decision-making parameters: Bayesian hierarchical expectation-maximization method

**Figure 3:**
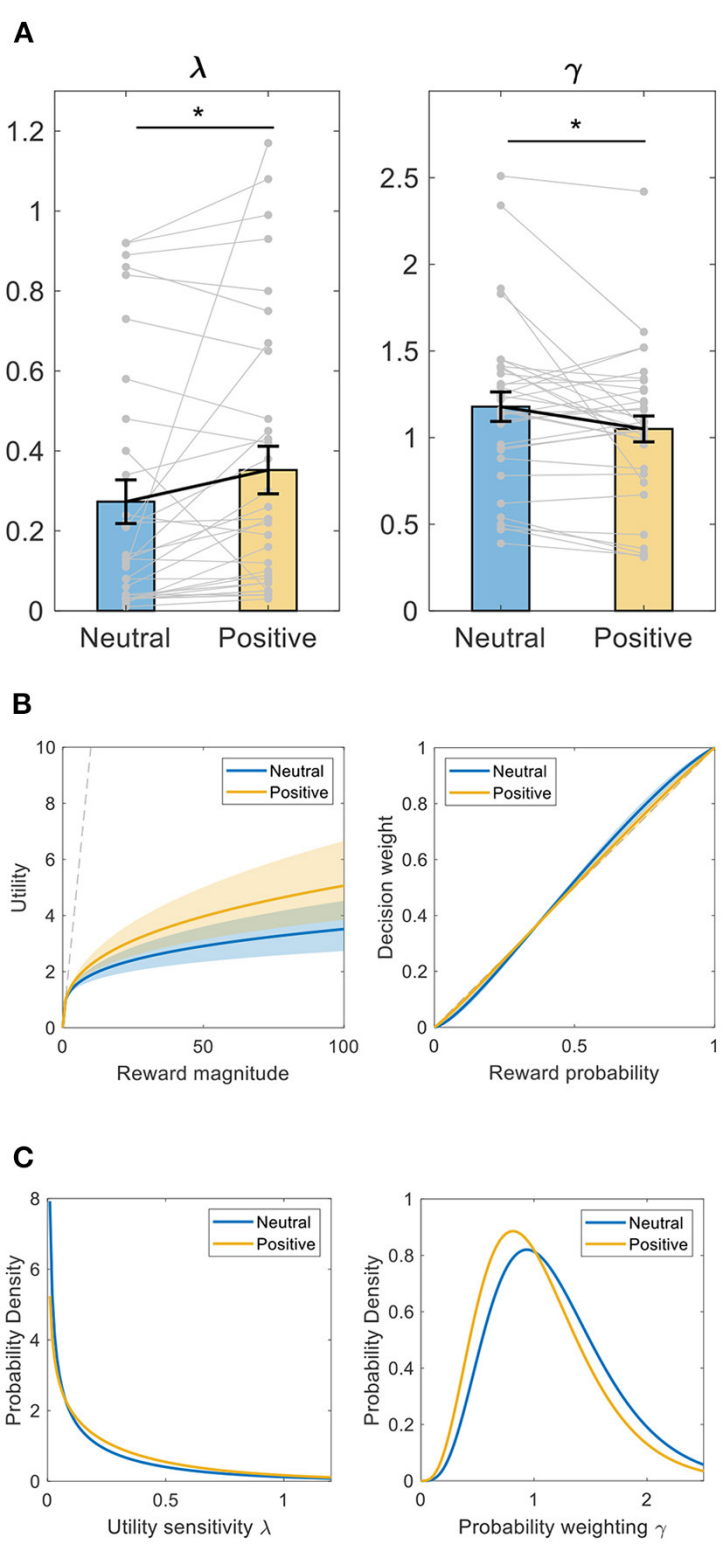
Intervention effect on decision-making parameters using Bayesian hierarchical expectation-maximization method. **(A)** utility sensitivity λ and probability weighting γ. *p < 0.05, Wilcoxon signed-rank test. Data shown as mean ± SE. **(B)** illustration of the utility and probability weighting function changes after positive memory retrieval, plot with the mean ± SE of each parameter. Left panel, utility (u) as a function of reward magnitude (x). Right panel: decision weight (w) as a function of objective probability (*p*). The dashed line represents linear utility or probability weighting. **(C)** the estimated population prior distributions for utility sensitivity λ and probability weighting γ.

We first fit the computational models to subjects' choices with a Bayesian hierarchical expectation-maximization method. As plotted in Figure 3A, between-intervention comparison showed that compared to after neutral memory retrieval, subjects had greater λ (Wilcoxon signed-rank test, *Z* = −2.457, *p* = 0.014, *d* = 0.354) and smaller γ (Wilcoxon signed-rank test, *Z* = −2.046, *p* = 0.041, *d* = 0.427) after positive memory retrieval. Greater λ indicates that subjects became less risk-averse after positive memory retrieval (Figure 3B, left panel). Since after positive autobiographical memory retrieval, subjects had a λ closer to 1 (Wilcoxon signed-rank test, *Z* = −1.278, *p* = 0.023, *d* = 0.354 based on the absolute value of the difference of λ from 1), this also indicates subjects became more risk-neutral or rational in utility sensitivity after positive memory retrieval.

Smaller γ indicates that subjects became either purely less S-shaped in probability weighting or more linear, objective in probability weighting (Figure 3B, right panel). The latter, however, was not supported by the data because subjects had a similar absolute value of the difference of γ from 1 (since γ = 1 indicates objective probability weighting) after positive vs. neutral memory retrieval (Wilcoxon signed-rank test, *Z* = −1.063, *p* = 0.288, *d* = 0.314). Therefore, compared to neutral memory retrieval, subjects became less S-shaped in probability weighting after positive memory retrieval. That is, they became more risk-seeking at small probabilities and more risk-averse at large probabilities.

We also plotted the estimated population prior distributions for λ and γ, respectively, in Figure 3C. Consistent with the above individual data, compared to after neutral memory retrieval, the prior distribution of λ shifted toward the right side or bigger values while that of γ shifted toward the left side or smaller values after positive memory retrieval.

### Decision-making parameters: Maximum likelihood

**Figure 4:**
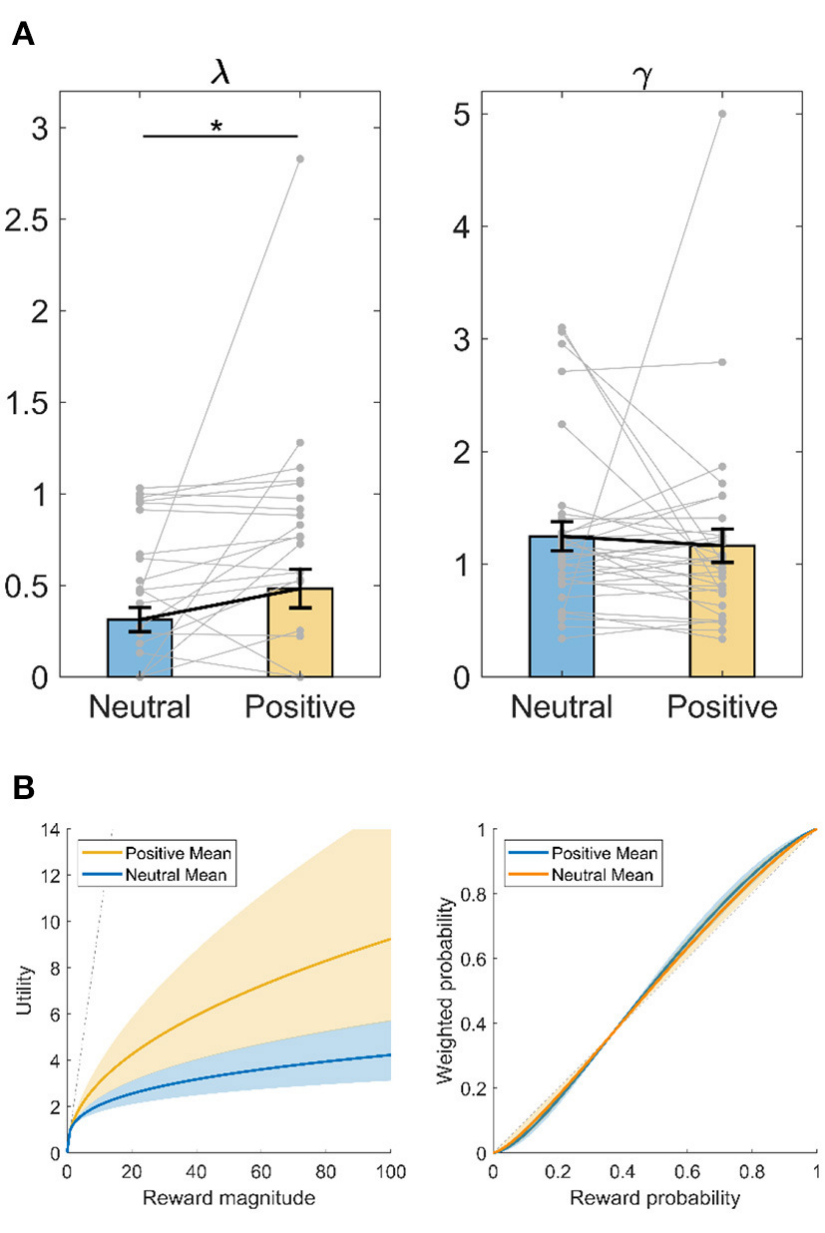
Intervention effect on decision-making parameters using maximum likelihood method. **(A)** Utility sensitivity λ and probability weighting γ. *p < 0.05, Wilcoxon signed-rank test. Data shown as mean ± SE. **(B)** Illustration of the utility and probability weighting function changes after positive memory retrieval, plot with the mean ± SE of each parameter. Left panel, utility (u) as a function of reward magnitude (x). Right panel: decision weight (w) as a function of objective probability (*p*). The dashed line represents linear utility or probability weighting.

We also fit the computational models to subjects' choices with a maximum likelihood method. As plotted in Figure 4A, compared to after neutral memory recall, subjects had greater λ (Wilcoxon signed-rank test, *Z* = −2.315, *p* = 0.021, *d* = 0.346) after positive memory recall. Greater λ indicates that subjects became less risk-averse after positive memory recall (Figure 4B, left panel). Since after positive memory recall, subjects had a λ closer to 1, this also indicates subjects became more risk-neutral or rational in utility sensitivity after positive memory recall. In contrast, there was no difference in probability weighting parameter γ after positive vs. neutral memory recall (Wilcoxon signed-rank test, *Z* = −0.884, *p* = 0.376, *d* = 0.080).

## Discussion

To our best knowledge, this is the first study that investigates the influence of positive autobiographical memory retrieval on decision-making under risk. This is also one of the few studies that investigate whether psychological, behavioral, or dietary interventions affect decision-making under risk [e.g., refer to (32) for a study of an internet-based cognitive behavioral therapy and self-report risk-taking behaviors in patients with generalized anxiety disorder, and (33) for a study of probiotics and decision-making with the Iowa Gambling Task in patients with Fibromyalgia]. Here, using both a Bayesian hierarchical expectation-maximization method with population-level priors and a maximum likelihood method without population-level priors, we identified a consistent effect of positive memory retrieval on utility sensitivity (λ), suggesting that subjects became more risk-neutral or rational in utility sensitivity after positive memory retrieval. Importantly, the change here is in the opposite direction compared to those reported in depressive disorders (34), seasonal affective disorder (35), and generalized anxiety disorder (14). These results suggest that positive autobiographical memory retrieval may have therapeutic effects for patients with these mental disorders. These results may have important clinical implications because these and similar decision-making deficits are resistant to clinical treatment and remain even when patients are in remission (36, 37).

In contrast, the effect of positive memory retrieval on probability weighting (γ) was present in the Bayesian hierarchical expectation-maximization fitting but absent in the maximum likelihood fitting. It raises the possibility that the influence of positive memory retrieval on probability weighting may be unreliable and requires further in-depth investigation. Altered probability weighting, specifically, S-shaped probability weighting with the tendency to underweight small probabilities and overweight large probabilities has been reported in patients with obsessive-compulsive and hoarding disorders (15) and people with high levels of depression (16). It will be interesting for future studies to confirm if positive memory retrieval helps alleviate such S-shaped probability weighting and has potential therapeutic effect.

As one subfield in positive psychology, positive emotions have been attracting much research interest (38, 39). For instance, positive emotions have been shown to broaden attention, increase cognitive flexibility, and enhance resilience (38, 39). As an essential strategy to increase positive emotions, positive autobiographical memory retrieval activates the brain reward system especially the striatum and the mPFC (17–19), buffers the hypothalamic-pituitary-adrenal axis response to acute stressors (19), and reduces delay discounting or impulsive choices (18). Adding to these findings, the present study suggests that positive autobiographical memory retrieval reduces risk-aversion. As mentioned in the introduction, one potential underlying neural mechanism of such effects might be the enhanced activation of the mPFC. The mPFC has been linked to risk processing and greater activation of the ventral mPFC is associated with higher risk-seeking (22). It remains for future neuroimaging studies to test if the mPFC mediate the effects of positive autobiographical memory retrieval reported here. Based on our findings and evidence reviewed above, habitual positive autobiographical memory retrieval may be employed as an important clinical interventional strategy for patients with depressive and anxiety disorders. People generally take photos of positive, important moments in everyday life, many further share those photos with others *via* social networking services. Those photos can be used as cues for positive memory retrieval to enhance stress coping and modify altered decision-making tendencies.

Our findings are consistent with previous reports that positive mood is associated with optimism about future events and risk-taking behaviors [for a review, (40)]. For instance, when in a happy mood, people tend to think positive events are more likely and negative events less likely (41). They are also more willing to pay for lotteries (42). One recent study has tried to elucidate the underlying cognitive and neural computational mechanism of this phenomenon and showed that task feedback-induced positive mood increases the weighting of potential gains while decreases the weighting of potential losses (43). The ventral mPFC and the anterior insula were found to mediate these effects, respectively. By focusing on reward alone and removing the influence of loss, the current study further showed that positive autobiographical memory retrieval reduces risk aversion, providing new cognitive computational explanations for the above phenomenon. In contrast to incident mood and task feedback used in previous studies, recalling positive autobiographical memories used in the present study has the potential to be employed as a therapeutic tool for managing altered decision-making under risk in patients with mental disorders.

Our study has several limitations. Firstly, to increase statistical power and remove the influence of age, we limited our subjects to those in their twenties. This, however, also refrains us from generalizing our findings to other age groups. Secondly, we tested only the immediate effect of positive autobiographical memory retrieval. How long the effect lasts is another important question to be answered by future studies. Thirdly, we investigated decision-making after positive autobiographical memory retrieval and used decision-making after neutral autobiographic memory retrieval as the control condition. That is, we did not conduct the same task at baseline before memory retrieval, which did not allow us to explicit show how each memory retrieval affects decision-making. The reason was that we used a crossover design and subjects had already performed the decision-making task twice, one after positive and the other after negative memory retrieval; including the decision-making task at baseline would be too demanding and effort-consuming for subjects. Fourthly, we focused on decision-making with reward only, and therefore our results may not be generalizable to decision-making with loss. Fifthly, to evaluate risk-based decision-making, we used a description-based task in which subjects were given explicit information on reward magnitude and probability. There is, however, another paradigm known as experience-based decision-making in which decision variables are not explicitly known and subjects had to learn those information based on trial-and-error experience. Recent studies suggest that people make inconsistent choices in description vs. experience based tasks, a phenomenon known as the “description-experience gap” (44, 45). It will be interesting for future studies to test if positive autobiographical memory retrieval affects decision-making in experience-based tasks. Sixthly, the effects we observed were only small to medium in size (i.e., *d* = 0.377 and 0.447). Since pictures are generally easier to recall than words (46), greater effects may be achieved by employing photographs of people's happy moments for memory retrieval. Seventhly, since it is fairly easy for subjects to notice the purpose of the study, we did not blind subjects about the purpose of the study and this might have caused some expectation bias. Nevertheless, we speculate that such bias is unlikely to be a concern here because it is generally hard for subjects to think of the influence of memory retrieval on decision-making, especially considering the fact that all of them were medical undergraduates and were not trained in relevant fields such as economics or cognitive psychology. Future studies are required to address these limitations and confirm and improve the findings reported in the present study.

## Data availability statement

The data that supports the findings of this study are available from the corresponding author upon reasonable request.

## Ethics statement

The studies involving human participants were reviewed and approved by Institutional Review Board of Yamaguchi University Hospital. The patients/participants provided their written informed consent to participate in this study.

## Author contributions

CC and SN: conceptualization. NS, YM, CC, KH, KM, and YO: methodology. TM: resources. NS, YM, and CC: formal analysis. NS, CC, KM, MH, and EO: investigation. NS and CC: writing—original draft preparation. All authors: writing—review and editing. All authors have read and agreed to the published version of the manuscript.

## Funding

This research was supported by grants from the Japan Society for the Promotion of Science (JSPS) KAKENHI (Grant Number 19K17063) and SENSHIN Medical Research Foundation to CC.

## Conflict of interest

The authors declare that the research was conducted in the absence of any commercial or financial relationships that could be construed as a potential conflict of interest.

## Publisher's note

All claims expressed in this article are solely those of the authors and do not necessarily represent those of their affiliated organizations, or those of the publisher, the editors and the reviewers. Any product that may be evaluated in this article, or claim that may be made by its manufacturer, is not guaranteed or endorsed by the publisher.

## Supplementary material

Supplementary Figure S1 A typical screen shown to a subject during the memory retrieval intervention. The upper line is the initial cue (“getting an acceptance letter”). The lower line is the scanned image of the subject's response record in day 1 autobiographical memory recall test.
