# Supplementary material for: The effect of positive autobiographical memory retrieval on decision-making under risk: A computational model-based analysis
Source: Front Psychiatry. 2022 Sep 6;13:930466. doi: 10.3389/fpsyt.2022.930466 (PMC9485606; doi:10.3389/fpsyt.2022.930466)
Supplement: Supplementary Figure S1 — A typical screen shown to a subject during the memory retrieval intervention. The upper line is the initial cue (“getting an acceptance letter”). The lower line is the scanned image of the subject's response record in day 1 autobiographical memory recall test. [file Image_1.pdf]

The effect of positive autobiographical memory retrieval on decision-making under risk: a computational model-based analysis

Supplementary material

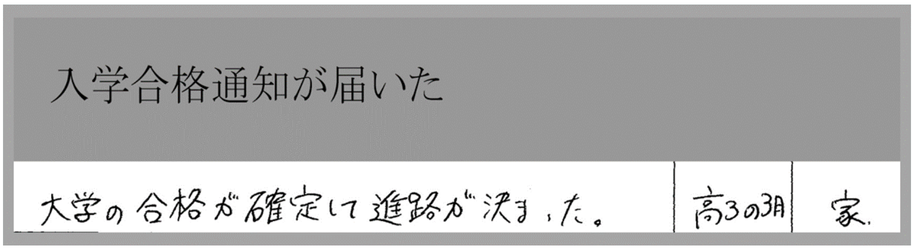

Figure S1. A typical screen shown to a subject during the memory retrieval intervention. The upper line is the initial cue (“getting an acceptance letter”). The lower line is the scanned image of the subject’s response record in day 1 autobiographical memory recall test.
